# Regulation of Endothelial Adherens Junctions by Tyrosine Phosphorylation

**DOI:** 10.1155/2015/272858

**Published:** 2015-10-18

**Authors:** Alejandro Pablo Adam

**Affiliations:** Center for Cardiovascular Sciences and Department of Ophthalmology, Albany Medical College, Albany, NY 12208, USA

## Abstract

Endothelial cells form a semipermeable, regulated barrier that limits the passage of fluid, small molecules, and leukocytes between the bloodstream and the surrounding tissues. The adherens junction, a major mechanism of intercellular adhesion, is comprised of transmembrane cadherins forming homotypic interactions between adjacent cells and associated cytoplasmic catenins linking the cadherins to the cytoskeleton. Inflammatory conditions promote the disassembly of the adherens junction and a loss of intercellular adhesion, creating openings or gaps in the endothelium through which small molecules diffuse and leukocytes transmigrate. Tyrosine kinase signaling has emerged as a central regulator of the inflammatory response, partly through direct phosphorylation and dephosphorylation of the adherens junction components. This review discusses the findings that support and those that argue against a direct effect of cadherin and catenin phosphorylation in the disassembly of the adherens junction. Recent findings indicate a complex interaction between kinases, phosphatases, and the adherens junction components that allow a fine regulation of the endothelial permeability to small molecules, leukocyte migration, and barrier resealing.

## 1. Introduction

Intercellular adhesion is a hallmark of all Metazoa. Complex organisms have evolved sophisticated methods to create adhesive forces that are strong enough to hold the organisms together but, at the same time, flexible enough to allow tissue remodeling and physiological adhesive changes. In particular, the adherens junction (AJ) is a multiprotein structure present in most organisms ranging from insects to mammals [1, 2]; its basic structure comprises transmembrane cadherins and cytosolic catenins linking the cadherin to the cytoskeleton [3]. All classical cadherins are composed of five extracellular domains (EC1–EC5), a single transmembrane domain, and a short cytoplasmic C-terminal tail. Trans-homodimerization occurs by the interaction of two EC1 domains of opposing cadherins [4]. VE-cadherin (cadherin 5) was discovered in the early 1990s [5, 6] and is a major component of endothelial cell-cell contacts. VE-cadherin is critical for endothelial biology and is required for vessel maturation in multiple species ranging from zebrafish [7] to mice [8, 9]. Similar to other classical cadherins, the cytoplasmic tail of VE-cadherin contains the binding regions for p120 catenin at the juxtamembrane domain (JMD) and for *β*-catenin or *γ*-catenin at the C-terminal catenin binding domain (CBD). Binding of p120 catenin stabilizes junctional cadherins by preventing cadherin endocytosis (reviewed in [3, 10]), while *β*-catenin associates with *α*-catenin, providing the link to the actin cytoskeleton [11, 12].

Endothelial cells play a critical role in the regulation of vasoreactivity, hemostasis, and leukocyte recruitment. Vascular endothelial cells are also critical for maintaining normal tissue function by acting as a selective barrier that regulates the passage of fluid, macromolecules, and leukocytes from the vascular space to the interstitium. The activation of proinflammatory pathways induces a loss of endothelial barrier function through activation of membrane receptors in endothelial cells, triggering several signaling cascades, including the activation of kinase signaling, small GTPase-mediated actin cytoskeleton remodeling, and calcium release (reviewed in [13–16]). Two pathways mediate the passage across the endothelial barrier. Endothelial cells may allow the transport of proteins and even cells through their cell body in what has been called the transcellular pathway. In this pathway, fluid and proteins are actively transported in a complex system of vesicles from the luminal to the basal side of the cell, where the vesicular content is released [13, 17]. Leukocytes have also been shown to migrate through single endothelial cells both* in vitro* and* in vivo* [18, 19]. In contrast, many proinflammatory mediators promote the disengagement of the AJ-based contacts, allowing the passage of fluids and leukocytes through a paracellular pathway, that is, between two endothelial cells. By regulating the paracellular pathway, VE-cadherin-based cell-cell contacts maintain the strong intercellular adhesion required for the vessel's barrier function, while at the same time allowing for sufficient plasticity when required. This review will focus on the regulation of the paracellular pathway by tyrosine kinase signaling, with special emphasis on discussing the findings that support and those that argue against a direct effect of cadherin and catenin phosphorylation in the disassembly of the adherens junction. Recent findings indicate a complex interaction between kinases, phosphatases, and the adherens junction components that allow a fine regulation of the endothelial permeability to small molecules, leukocyte migration, and barrier resealing.

## 2. Intercellular Adhesion Is Regulated by Phosphorylation of Cadherins and Catenins

The development of antibodies that recognize phosphotyrosine residues quickly enabled research that demonstrated a critical role for tyrosine phosphorylation in the modulation of intercellular adhesion, in particular through the regulation of AJ-based contacts. Maher et al. were the first to show that cell-cell junctions in epithelial cells (PtK2 and MDCK) and chicken embryo fibroblasts contained proteins phosphorylated on tyrosine [20]. Within the following decade, it became very clear that treatment of cells with pervanadate (a pan-specific tyrosine phosphatase inhibitor) or oncogene-induced transformation in cell culture can induce a dramatic increase in the phosphotyrosine content at the cell junctions by increasing the phosphorylation of VE-, N- and E-cadherin, as well as *α*-catenin, *β*-catenin, and *γ*-catenin [20–32]. A similar observation was made in rats, in which an intravenous injection of sodium orthovanadate increased the junctional staining of an anti-phosphotyrosine antibody in the intestine, heart, and liver [23]. Further, EGF treatment in human epidermoid carcinoma A431 cells induced *β*-catenin and *γ*-catenin phosphorylation [33], demonstrating that endogenous kinases could also promote catenin phosphorylation in response to growth factors. At the same time, Reynolds et al. [34] described a 120 kDa protein that was highly phosphorylated on tyrosine in v-Src transformed chicken embryo cells, whose identity was later found to be the catenin family member p120 [35] and to associate with E-cadherin [36]. Src was then shown to be able to phosphorylate multiple tyrosines at the amino terminus of p120 [37]. Early research unambiguously demonstrated that tyrosine phosphorylation can disrupt cadherin-based adhesions. Short-term pervanadate treatment increased phosphotyrosine content at MDBK cell junctions, while long term treatment disrupted cell adhesion [22]. Using v-Src as a model, it was shown that oncogene-driven overactivation of tyrosine kinases promoted a loss of intercellular adhesion in a number of epithelial and fibroblast cells [21, 24–28, 30, 38].

**Figure 1:**
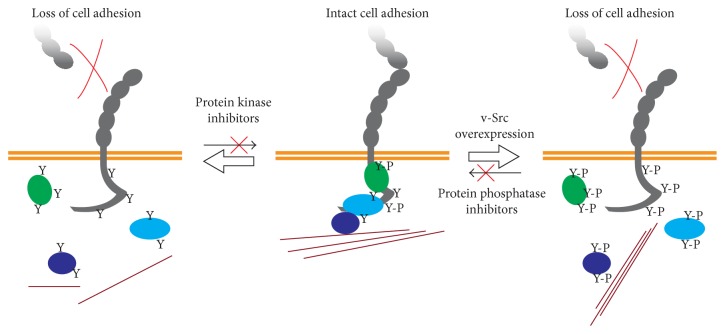
Adherens junction-based cell adhesion requires a tight balance of tyrosine kinases and phosphatases. Oncogenic Src signaling and blockade of phosphatase activity, as well as complete inhibition of kinase activity, can lead to AJ disruption and loss of cell-cell adhesion.

Oncogenic Src mutants may have different substrate specificity than endogenous kinases [39], which may lead to unintended consequences in cells overexpressing v-Src. Indeed, it soon became clear that not all tyrosine kinase activity led to the disruption of the adherens junctions and that phosphorylation in tyrosine could also mediate junctional stability [40–46]. While massive phosphorylation caused by phosphatase inhibition or v-Src overexpression induces dramatic changes in cell adhesion strength, inhibition of protein tyrosine kinase (PTK) signaling can also lead to disrupted cell adhesion (Figure 1). In MCF7 human mammary adenocarcinoma cells, a delicate balance of Src activity was required for maintaining normal adherens junction integrity, since either blocking Src activity (via dominant negative Src constructs or pharmacological inhibition using PP2) or overactivation of Src (by expression of the constitutively active mutant Y530F Src) induced a marked junctional disruption [47]. These results suggest that the effects of Src-mediated signaling in the regulation of the adherens junctions strictly depend on the level of activity and that while high levels of Src signaling disrupt intercellular adhesions, low basal levels of Src are required for normal cellular adhesion. These findings led the authors to propose that the loss of cell-cell adhesion observed in gain-of-function studies using oncogenic v-Src constructs may reflect events of cell transformation and epithelial to mesenchymal transition, while the role of basal endogenous SFKs promoting the strengthening of cell adhesion reflected a physiological role in AJ maintenance [48]. In that regard, it was shown that, in mouse keratinocytes, p120, *β*-catenin, and *γ*-catenin (but not E-cadherin) tyrosine phosphorylation was increased after calcium-induced differentiation, which coincided with an increased association of *α*-catenin and p120 with E-cadherin [40]. Conversely, addition of the kinase inhibitors genistein, tyrphostin, or PP1, or Fyn deficiency, diminished cell-cell adhesion in a dispase-mediated cell release assay and mice deficient for Fyn and Src displayed deficient cell-cell adhesion in skin [40]. Similarly, PP2 or genistein treatments reduced N-cadherin-based adhesion in Rat-2 fibroblasts, an effect that was attributed at least in part to a requirement for cortactin phosphorylation to sustain N-cadherin adhesion [41]. Src activity is also required to maintain junctional stability in* Drosophila*, as a dominant negative mutant of the Src homolog Src42A induced the disorganization of DE-cadherin contacts [42] and* Drosophila* embryos lacking both Src homologs Src42A and Src64 showed diminished DE-cadherin and armadillo staining at cell-cell junctions [43]. Together, these findings demonstrate that tyrosine kinase signaling can lead to AJ formation and stability.

The mechanisms involved in Src-mediated AJ formation are not well understood. Tyrosine kinases may promote AJ assembly through phosphorylation of its components or indirectly via the regulation of tyrosine phosphatases as well as small GTPases. For example, Abl kinases can promote AJ formation through the regulation of Rho and Rac signaling [44, 45], while in E8 chicken retina cells, p120-associated Fer is essential to maintain *β*-catenin binding to N-cadherin, by promoting Y152 phosphorylation in the phosphatase PTP1B, which in turn was responsible for dephosphorylating Y654 in *β*-catenin [46]. PTK signaling can thus mediate both assembly and disassembly of adherens junctions in a complex interplay. Understanding exactly how and when a phosphorylation event will lead to loss or strengthening of cell adhesions is one of our main challenges ahead.

## 3. Cadherins Also Can Be Upstream of Tyrosine Kinases and Regulate RTK Signaling

**Figure 2:**
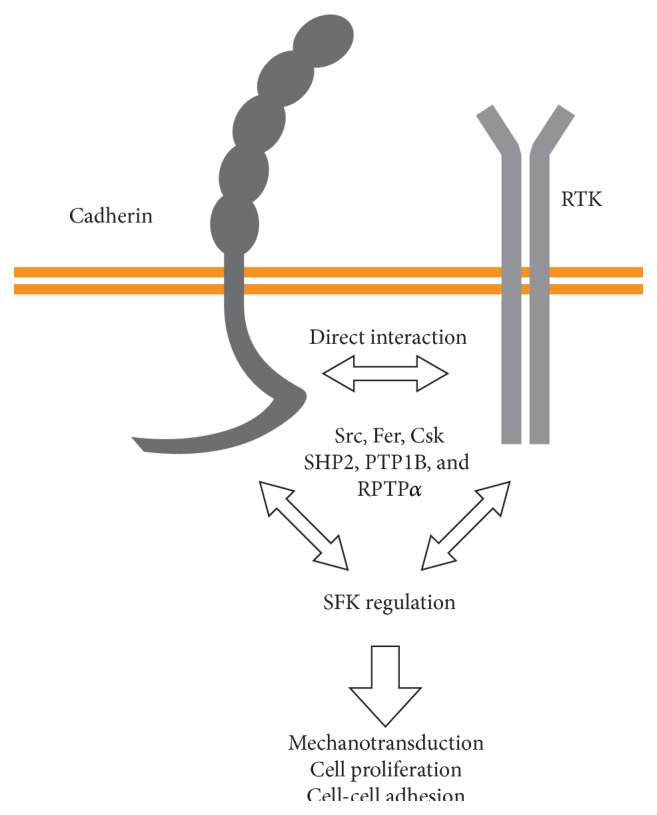
Cadherins can regulate PTK activity. The proposed mechanisms include the direct modulation of kinases and phosphatases as well as interactions with receptor tyrosine kinases. Cadherin-mediated PTK activity has been shown to be involved in mechanosensory transduction, contact inhibition of cell proliferation, and strength of cell adhesion.

The research discussed so far has placed cadherins and catenins downstream of PTK activity, but E-cadherin engagement can also regulate PTK activity, placing cadherins correspondingly upstream of these kinases (Figure 2). For instance, preventing E-cadherin engagement in MCF7 cells using E-cadherin blocking antibodies reduced the amount of active Src at cell-cell junctions, while beads coated with E-cadherin/Fc chimera promoted a rapid increase in active Src [47]. The mechanism by which E-cadherin activates Src signaling was found to depend on the tyrosine phosphatase RPTP*α* [49–51], presumably by removing the phosphate at the Src autoinhibitory tyrosine 530 [52]. Cadherins also have been shown to bind and modulate receptor tyrosine kinase (RTK) signaling, both positively and negatively [53]. E-cadherin-mediated cell adhesion inhibited EGFR signaling in MDCK cells [54] but induced ligand-independent EGFR activation leading to increased Erk signaling in HaCat keratinocytes [55] and in mammary epithelial cells [56]. N-cadherin, on the other hand, was not found to be associated with EGFR [54]. However, N-cadherin engagement stimulated neurite outgrowth in cerebellar neurons through the activation of FGFR [57], a pathway that was later found to promote tumor metastasis [58, 59]. Similar interactions between VE-cadherin and VEGFR2 are required for contact inhibition of endothelial cell growth [60] and for the endothelial response to shear stress [61–63]. Further, VEGF-induced Src activation required the dissociation of Csk (a kinase that inhibits SFK activation [64]) from VE-cadherin and the recruitment of SHP2, which then dephosphorylated Src at tyrosine 530, allowing its activation [65]. This mechanism is reminiscent of the E-cadherin/RPTP*α*-induced Src activation in MCF7 cells [47, 49–51]. Conversely, p120 overexpression in HUVECs blocked neutrophil TEM through preventing ICAM-1-induced VE-cadherin phosphorylation and the association of VE-cadherin with active (pY419) Src [66, 67], placing p120 association with VE-cadherin upstream, rather than downstream, of Src activation at least in the context of neutrophil transendothelial migration. Together, these results show that cadherins can either promote or prevent RTK-mediated signaling.

Although some of the molecular mechanisms are being teased out, it still remains largely unknown how cadherin association can regulate RTK signaling. An important clue comes from the VE-cadherin and VEGFR2-dependent response to shear in endothelial cells [61–63]. Fluid shear force is transmitted by PECAM-1, leading to VE-cadherin-dependent activation of VEGFR2 and Akt signaling [63]. The mechanism involves an increase in PECAM-1 tension, triggering PECAM-1/vimentin association, and a reduction in the levels of VE-cadherin tension [68]. It is possible that tension-mediated changes in cadherins may not be limited to VEGFR2 activation under shear. Cadherins are constantly under tension [12, 68, 69] and it was recently shown that tension at VE-cadherin junctions can regulate cell-cell contacts [70, 71]. It is not known, however, whether changes in tension at the AJ can modulate other RTK responses or cadherin phosphorylation itself.

## 4. The Adherens Junction Is Phosphorylated in the Endothelium

Similar to other classical cadherins, the phosphorylation state of VE-cadherin was found to be associated with differences in endothelial function. Lampugnani et al. first showed that a phosphotyrosine antibody labeled endothelial cell-cell contacts [72] by showing that loosely confluent HUVECs displayed strong junctional phosphotyrosine labeling, while this staining was reduced in tightly confluent cultures. Further studies demonstrated that VEGF induced an increase in endothelial permeability that required tyrosine kinases [73] and that VEGF promoted the phosphorylation of tyrosines in VE-cadherin, *β*-catenin, *γ*-catenin, and p120 [74]. VEGF also stimulated the dephosphorylation of p120 serine residues [75]. Most importantly, inhibition of SFK activity prevented edema formation in several animal models [76–79], demonstrating a causal role for SFKs in VEGF-induced loss of endothelial barrier function.

The particular phosphorylation sites of VE-cadherin in response to VEGF are a matter of intense investigation. This question became further complicated by the lack of specificity of some commercial antibodies used in previous studies. Wallez et al. [80] showed that VEGF-165 induces VE-cadherin phosphorylation at tyrosine 685 in HUVECs, as measured by phosphopeptide mapping.* In vitro*, Src was able to directly phosphorylate a short peptide containing this residue. Other peptides containing C-terminal tyrosines (from Y645 to Y784) were not a good substrate for this* in vitro* assay, suggesting that either Src is not capable of directly phosphorylating these tyrosines in VE-cadherin or that other docking site(s) in the full length protein are needed for this reaction. No phosphorylation in serine was detected upon VEGF treatment [80]. This is in sharp contrast with the findings by Gavard and Gutkind [81], who showed that VEGF induces VE-cadherin phosphorylation at serine 665 in HUVECs, a key step to promote *β*-arrestin binding and VE-cadherin endocytosis. VE-cadherin serine 665 phosphorylation was also implicated downstream of R-Ras, a small GTPase required for vascular differentiation that is downregulated in the leaky tumor vasculature [82]. In HUVECs, expression of an active form of R-Ras (R-Ras38V) prevented VEGF-induced phosphorylation at S665 and VE-cadherin endocytosis, without affecting VEGF-induced tyrosine phosphorylation at Y658 or Y731 [83].

VE-cadherin phosphorylation also occurs in response to other stimuli, including TNF-*α*, LPS, H_2_O_2_, and high glucose [84–87], albeit at slower kinetics than after VEGF treatment. Addition of TNF-*α* [84] or LPS [85] to human lung microvascular endothelial cells (HMVEC-L) induced a sustained loss of barrier function, together with VE-cadherin tyrosine phosphorylation. This phosphorylation, however, was only detectable in cells treated with orthovanadate and phenylarsine oxide, while TNF-*α*- or LPS-induced increase in permeability did not require phosphatase inhibition, raising the question of whether endogenous phosphatases are sufficient to blunt the tyrosine phosphorylation induced by these agonists, without affecting the increase in permeability. In any case, a requirement of tyrosine kinase activity was demonstrated by the ability of several kinase inhibitors to block the increase in monolayer permeability induced by TNF-*α* or LPS [84, 85]. Notably, nonspecific inhibitors such as genistein, herbimycin A, and geldanamycin were much more efficient at preventing TNF-*α*-induced loss of barrier function than SFK-specific inhibitors PP1 and PP2, suggesting that other kinases may be involved in parallel pathways [84].

A wealth of data unmistakably points to a very important role for SFK-mediated tyrosine phosphorylation of endothelial AJ in the regulation of barrier function. However, as in the case of epithelial cells presented above, the link is not straightforward or unidirectional. SFK activity and VE-cadherin phosphorylation can be observed* in vivo* in the absence of any pathological condition. Lambeng et al. [88] showed that VE-cadherin is highly phosphorylated in some tissues of healthy adult mice, particularly lung and uterus, and that VE-cadherin phosphorylation increased upon angiogenic stimuli. More recently, it was shown that VE-cadherin phosphorylation at tyrosine 685 in mouse ovary and uterus varied throughout the estrous cycle [89]. Moreover, Orsenigo et al. [90] showed that venules and capillaries, but not arterioles, in mouse bladder and diaphragm display constitutive VE-cadherin phosphorylation at tyrosines 658 and 685 and that this phosphorylation is dependent on basal SFK activity in venules. In rats, carotids showed much lower VE-cadherin phosphorylation at tyrosine 685 than jugular veins. Interestingly, a jugular bypass to expose the vein to arterial bloodstream drastically reduced VE-cadherin phosphorylation [90]. More recently, Wessel et al. [91] showed that tyrosine 731, but not tyrosine 685, was constitutively phosphorylated in mouse lungs. Substitution of wild-type VE-cadherin for Y685F mutant, but not Y731F mutant, resulted in an attenuation of the dermal vascular leakage after injection of VEGF or histamine. A similar knock-in strategy was used by Sidibé et al., who found that Y685F VE-cadherin mice displayed increased vascular leakage in the uterus and ovary, suggesting that VE-cadherin phosphorylation at this site may have an important role maintaining vessel integrity [92]. In contrast to the observations by Orsenigo et al. [90], Wessel et al. [91] did not find that VE-cadherin was constitutively phosphorylated at tyrosine 685 in the venules of the cremaster vasculature. However, treatment of mice with pervanadate promoted tyrosine 685 phosphorylation in venules, suggesting that differences in basal tyrosine phosphatase activity (either due to differences in the cremaster vasculature or more general mouse strain differences) could explain the difference between these two reports. Strikingly, the pervanadate treatment was unable to promote an increase in VE-cadherin phosphorylation in arterioles, which the authors attributed to a possible lack of active kinases in the vicinity of VE-cadherin [91]. Tyrosine 685 has been proposed to be the binding site for Csk and Y685F VE-cadherin mutant does not associate with Csk [65, 93], raising the possibility that differential Csk association could regulate the access of active kinases to VE-cadherin C-terminal tyrosines. Alternatively, catenin binding may be involved in the regulation of VE-cadherin phosphorylation. For example, p120 overexpression can reduce the association of VE-cadherin to active Src [66, 67], while it may recruit Fer and PTP1B to the AJ as shown in retina cells [46]. A potential role for catenins regulating VE-cadherin phosphorylation in venules remains untested. In all, these findings show not only that VE-cadherin phosphorylation at particular tyrosines is an important step in the loss of endothelial cell-cell adhesion leading to an increase in permeability and TEM, but also that VE-cadherin can be phosphorylated in the absence of vascular leakage, demonstrating* in vivo* that other signals must be activated concurrently.

*In vitro*, the effect of SFK activation on human dermal microvascular cells depends on the method of activation [94]. Consistent with the findings in epithelial cells, overexpression of a constitutively active form (Y530A) of Src promoted VE-cadherin phosphorylation, monolayer gap formation, and loss of TEER. However, activation of endogenous SFKs by blocking Csk increased VE-cadherin phosphorylation without promoting an increase in monolayer permeability [94], demonstrating that while SFK activity may be required for the hyperpermeability induction by VEGF and other mediators, SFK activation alone is not sufficient to induce a loss of barrier function. Instead, SFK-induced AJ phosphorylation may act as a gatekeeper that allows edemagenic stimuli to promote an increase in permeability. In fact, bradykinin was able to induce vascular leakage on venules that displayed increased Src and VE-cadherin phosphorylation, but not in sites with low basal tyrosine phosphorylation [90]. Interestingly, the phosphorylation at tyrosine 685 in trachea venules quickly disappeared after bradykinin or histamine injections.* In vitro* assays suggested that this dephosphorylation event was due to clathrin-dependent VE-cadherin endocytosis and ubiquitin-mediated degradation, rather than a direct action of a phosphatase [90].

## 5. Leukocyte Transendothelial Migration Requires Multiple Tyrosine Phosphorylation Steps

Leukocyte infiltration into inflamed tissues is a major aspect of the body's response to damage. To arrive at the required location, leukocytes must travel through the endothelium, in a process called extravasation. This is a multistep process that involves complex interactions between the leukocyte and the endothelial cell. Leukocytes bind to activated endothelium and initiate a cascade of intermolecular contacts that allow them to traverse from the bloodstream into the stroma through the endothelium via either a transcellular route (i.e., through an endothelial cell) or a paracellular route (opening a gap between two adjacent ECs) (for reviews, see [95–97]). The endothelial response to leukocyte adhesion and migration involves the activation of multiple signaling pathways, notably Ca^2+^ release, Rho activation, actin remodeling, and tyrosine kinase activation, surrounding the leukocyte in what is called the adhesion cup and promoting the cytoskeletal changes to make room for the transmigrating leukocyte. This review will focus on tyrosine kinase signaling, and the reader is referred to recent excellent reviews [16, 98, 99] for a comprehensive discussion of all other known players involved.

**Figure 3:**
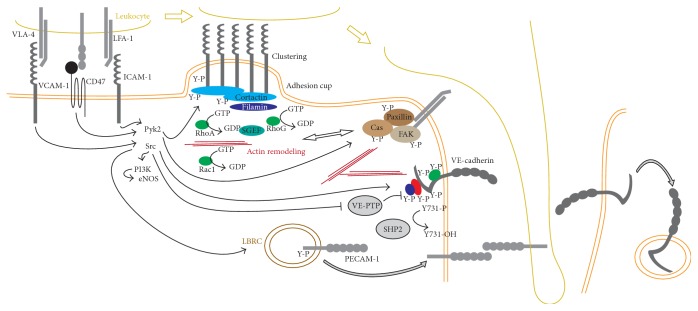
Simplified endothelial signaling cascades in response to leukocyte attachment. Adhesion of leukocytes through multiple transmembrane proteins such as ICAM-1, VCAM-1, and CD47 promotes activation of small GTPases (depicted as Rac1, RhoA, and RhoG) and PTK signaling, such as activation of Src and Pyk2. PTK activity leads to phosphorylation of actin binding proteins (e.g., cortactin) and focal adhesion components (FAK, paxillin, and Cas) that together with filamin promote ICAM-1 clustering and actin remodeling that is required for the formation of the adhesion cup. PTKs also promote the phosphorylation of VE-cadherin, which together with VCAM-1-mediated dissociation of VE-PTP from VE-cadherin leads to junctional hyperphosphorylation. At the same time, SHP2 mediates VE-cadherin dephosphorylation specifically at tyrosine 731. VE-cadherin endocytosis may follow. Src-mediated phosphorylation of PECAM-1 is required from PECAM-1 translocation from the LBRC to the plasma membrane.

Early on, a critical role was recognized for tyrosine phosphorylation in leukocyte transendothelial migration (TEM), at least in part mediated by leukocyte integrins binding to ICAM-1, leading to remodeling of the actin cytoskeleton (Figure 3). ICAM-1 ligation induces the tyrosine phosphorylation of multiple proteins, including focal adhesion kinase (FAK), paxillin, Cas [100], and cortactin [101]. ICAM-1 antibody-coated beads promoted the association of Src and tyrosine phosphorylated cortactin to ICAM-1. Inhibition of SFK activity prevented cortactin phosphorylation but not association with ICAM-1. Consistent with a model in which phosphorylated cortactin is required for ICAM-1 clustering, PP2 treatment significantly reduced the ability of fixed THP-1 monocytes to bind to activated HUVECs and prevented ICAM-1 clustering around the adhered cells [101]. Similarly, cortactin knockdown abolished PMN transmigration through TNF-*α*-activated HUVECs, which could be rescued by reexpression of wild-type cortactin-GFP, but not by a cortactin mutant in which three tyrosines (Y421, Y466, and Y482) were mutated to phenylalanine (cortactin 3F-GFP) [102]. ICAM-1 cross-linking-induced formation of actin stress fibers in TNF-*α*-treated HUVECs was also blocked by cortactin knockdown, PP2 treatment, or expression of either tailless ICAM-1-GFP or cortactin 3F-GFP. More importantly, cortactin siRNA blocked the clustering of actin and ICAM-1 around adherent PMN [102]. Altogether, these findings strongly argue for a critical role for SFK-mediated cortactin phosphorylation regulating ICAM-1 clustering and TEM. The definitive proof that cortactin mediates TEM* in vivo* was provided by Schnoor et al. [103] who found that loss of cortactin in mice reduced neutrophil recruitment. Cortactin knockout mice showed increased leukocyte rolling velocities, which was associated with a reduced adhesion to postcapillary venules and diminished ICAM-1 clustering around neutrophils. The mice also showed increased basal vascular leakage, thus mechanistically separating the regulation of barrier function from TEM.* In vitro*, an EPAC-specific cAMP analog rescued the increased permeability, while TEM efficiency was restored by expression of a constitutive form of RhoG [103], a GTPase that is activated downstream of ICAM-1 and Src by the SH3-containing guanine-nucleotide exchange factor (SGEF), a Rho-specific exchange factor [104].

Consistent with a role for cortactin-mediated ICAM-1 clustering, apical ICAM-1 mobility was reduced in HUVECs after ICAM-1 antibody-mediated cross-linking in cells expressing full length ICAM-GFP, but not tailless ICAM-GFP [102]. However, expression of an ICAM-1 deletion mutant lacking the intracellular tail was much more effective at preventing transcellular than paracellular TEM [105]. The implications of this finding are not completely clear, as ICAM-1 clustering via association with the actin cytoskeleton appears to be a critical component of the response to leukocyte binding regulating both paracellular and transcellular migration. ICAM-1 mobility was also reduced at the sites of ICAM beads binding to HeLa cells expressing wild-type ICAM-1-GFP, but not a C-terminal tail deletion [106]. ICAM-1 bead adhesion to HUVECs was prevented by inhibitors of Rac1, actin polymerization, or myosin II. Interestingly, MEFs from Src, Yes, and Fyn (SYF) triple SFK knockout mice reexpressing or not Src displayed similar ICAM-1-GFP FRAP kinetics and bead binding, suggesting that at least in MEFs SFK activity is not a major player in ICAM-1 dynamics [106].

Interestingly, leukocyte receptors can be phosphorylated on tyrosine as well (Figure 3). Src was shown to phosphorylate ICAM-1* in vitro* on tyrosine 512 [107]. Binding of activated HUVECs to fibrinogen induced the tyrosine phosphorylation of ICAM-1 and promoted ICAM-1/SHP2 interaction through a mechanism that required tyrosine 512 phosphorylation [107]. A possible role for this phosphorylation in ICAM-1 function was proposed because TNF-*α*-induced ICAM-1 cleavage was abolished by Y512A mutant [108]. In HUVECs, ICAM-1-mediated Src and eNOS activation was dependent on ICAM-1 tyrosine phosphorylation, because expression of a mouse Y518F mutant ICAM-1 construct (corresponding to the human Y512 residue) blocked ICAM-1 cross-linking-induced Src, eNOS, and Akt phosphorylation [109]. Importantly, this Y518F ICAM-1 construct was not as efficient as wild-type ICAM-1 in promoting PMN TEM in HUVECs. PP2 inhibition experiments showed that SFK activity was required for ICAM-1, Akt, and eNOS phosphorylation, while the PI3K inhibitor wortmannin was able to block eNOS, but not ICAM-1 phosphorylation, suggesting that PI3K acted downstream of Src and upstream of eNOS [109]. However, expression of Y512F ICAM-1 was almost as effective at promoting lymphocyte migration as wild-type ICAM-1 in GP8/3.9 immortalized rat brain microvascular endothelial cells [110], suggesting that this Src substrate site was not critical for ICAM-1 function. Further, endogenous ICAM-1 was not phosphorylated after incubation with lymphocytes in these cells [110]. Elucidation of the role of ICAM-1 phosphorylation in leukocyte transmigration will require further research, especially* in vivo*.

PECAM-1 is another leukocyte receptor known to be phosphorylated on at least two tyrosines, Y633 and Y686 [111]. Contrary to most leukocyte-interacting proteins, PECAM resides in a specialized compartment, named the lateral border recycling compartment (LBRC) [96]. Tyrosine phosphorylated PECAM is enriched in the LBRC [112] and this phosphorylation appears to be important for successful TEM, because either PP2 treatment [112] or mutation of tyrosine 633 [111] prevented PECAM recycling to the cell surface and TEM (Figure 3).

To allow TEM via the paracellular route, the endothelial cell must disassemble the adherens and tight junctions that sustain the strong homotypic intracellular contacts in order to create the space for their migration (Figure 3). Early work showed that leukocytes transmigrate through endothelial gaps* in vivo* [113] and* in vitro* [114] and that monocytes and U937 cells induced the reversible loss of junctional VE-cadherin and catenins during TEM [115]. There has been considerable interest in understanding how tyrosine phosphorylation affects the stability of adherens junctions in the context of TEM. Antibody-mediated ICAM-1 ligation promoted VE-cadherin phosphorylation in GPNT rat brain endothelial cells and bEND5 mouse brain endothelioma cells [116] and bead-mediated ICAM-1 cross-linking induced a rapid Src- and Pyk2-dependent phosphorylation of VE-cadherin in TNF-*α*-treated HUVECs [66, 117]. In particular, ICAM-1 ligation-induced, Src-mediated VE-cadherin phosphorylation can be blocked by p120 overexpression [66]. Similar phosphorylation events were observed after adhesion of monocytic THP-1 cells to TNF-*α*-treated HUVECs [117] and binding of rat peripheral lymph node lymphocytes to GPNT cells [116]. Tyrosine phosphorylation of catenins, ZO-1, or occludin was not detected after ICAM-1 cross-linking in GPNT cells [116]. Other receptors may activate similar downstream pathways as well, as cross-linking of CD47 in the endothelium induced Src, Pyk2, and VE-cadherin phosphorylation in activated HUVECs [118] and VCAM-1 cross-linking promoted VE-cadherin and VE-PTP dissociation in bEnd5 cells [119, 120]. In HUVECs, active pY419 Src and pY402 Pyk2 labeling showed a similar pattern surrounding the ICAM-1 beads, but neither *β*-catenin nor VE-cadherin was seen at sites of ICAM-1 engagement [117]. Nevertheless, treatment with the SFK inhibitor PP2 or expression of the Pyk2 dominant negative CRNK reduced the pY658 and pY731 signals and prevented neutrophil TEM. Further confirming a role for these two phosphorylated tyrosines, overexpression of Y658F and Y731F nonphosphorylatable VE-cadherin-GFP mutants strongly reduced paracellular TEM compared to wild-type VE-cadherin-GFP [117]. Surprisingly, VE-cadherin phosphorylation after ICAM-1 cross-linking in GPNT cells was insensitive to PP2 treatment, ruling out SFKs as the main kinases involved in this phosphorylation [116]. The reason for this discrepancy is unknown, but the differential localization of Src and other kinases in response to bead- or IgG-induced ICAM-1 cross-linking could potentially explain this conflicting result. To determine the specific sites of VE-cadherin phosphorylation in response to ICAM-1 ligation, CHO cells were engineered to express ICAM-1 together with either wild-type or mutant VE-cadherin-GFP. Tryptic digestion of ^32^P-labeled CHO-ICAM-1 cells showed that ICAM-1 cross-linking promoted VE-cadherin phosphorylation at Y731. Interestingly, the authors mentioned that the majority of phosphorylation events in VE-cadherin occur at serine and threonine, rather than tyrosine residues [116], but the effect of these phosphorylated residues remains unknown. To test the causal role of VE-cadherin phosphorylation, a series of Y/F point mutants was expressed in endothelioma cells derived from VE-cadherin null mice. Surprisingly, reexpression of wild-type VE-cadherin or a VE-cadherin-GFP fusion construct increased twofold, rather than decreased, TEM of antigen-specific T cells. Expression of the different mutants in CHO cells suggested that tyrosine 731 is the main phosphorylation site involved in TEM. When compared to wild-type VE-cadherin-GFP, expression of Y731F-VE-cadherin-GFP mutant allowed only 50% of TEM without affecting T-cell adhesion, while cells expressing Y658F and Y685F mutants allowed similar levels of TEM as wild-type VE-cadherin. A role for Y731* in vitro* was confirmed in GPNT cells, which express endogenous VE-cadherin. In these cells, expression of Y731F (as well as Y645F and Y733F, but not Y/F mutations in Y658, Y685, Y725, or Y757) significantly reduced T-cell TEM. No mutant affected lymphocyte adhesion to these cells [116]. Critically, mice harboring a Y731F knock-in mutation in VE-cadherin displayed drastically diminished leukocyte infiltration, thus directly demonstrating a crucial role for this tyrosine in leukocyte TEM* in vivo* [91]. While this tyrosine appears to be constitutively phosphorylated, leukocyte attachment induced its dephosphorylation through a mechanism that involved the phosphatase SHP2. It is yet unknown whether tyrosine 658 is also required* in vivo*, as suggested by the data obtained by Allingham et al. [117]. A similar knock-in approach might help answer this question. Thus,* in vitro* data show that leukocyte attachment can promote VE-cadherin phosphorylation, but* in vivo* experiments suggest that the critical VE-cadherin tyrosine is constitutively phosphorylated, suggesting that the main target for SFK-mediated phosphorylation downstream of leukocyte attachment (through at least ICAM-1, VCAM-1, and CD47 engagement) may be other proteins than VE-cadherin, such as cortactin, FAK, or eNOS.

## 6. The Case for Tyrosine Phosphorylation Regulating Cadherin/Catenin Association

Catenin binding is essential to support cadherin-based adhesions. Accordingly, p120 binding increases E-cadherin lateral clustering and adhesion strength [121, 122] and prevents endocytosis of E-cadherin [123, 124] and VE-cadherin [125–129]. *β*-catenin also increases VE-cadherin adhesion strength [130] and functions as a bridge to connect cadherins to *α*-catenin and thus the actin cytoskeleton [11, 12]. A critical role for this latter association was demonstrated by the expression of locked cadherin constructs that are fused directly to *α*-catenin and are thus independent of *β*-catenin association and dissociation. Expression of an E-cadherin construct fused to *α*-catenin promoted strong cell-cell adhesion [131, 132]. Moreover, cells expressing this construct were resistant to dissociation induced by pervanadate treatment [132]. Elegant studies by Schulte et al. [133] using knock-in mice expressing VE-cadherin-*α*-catenin chimera demonstrated that the dissociation of *α*-catenin from VE-cadherin is a required step for the induction of vascular permeability by VEGF or histamine and for allowing neutrophil or lymphocyte recruitment into inflamed tissues.

The observation that tyrosine phosphorylation regulates cell-cell adhesion, together with the finding that cadherins and catenins are targets for tyrosine kinases, led to intense research to determine how phosphorylation affected the adherens junction structure, with the notion that the phosphorylation of cadherins and/or catenins may lead to changes in cadherin/catenin association. Consistently, *β*-catenin phosphorylation may function as a switch to allow or prevent cadherin association with the actin cytoskeleton by affecting its ability to bind E-cadherin and *α*-catenin. In F9 cells, a phosphomimetic Y142E mutation in *β*-catenin did not coimmunoprecipitate with *α*-catenin, while Y654E *β*-catenin mutation reduced but did not abolish the ability to coimmunoprecipitate with E-cadherin [134], suggesting that these two tyrosines are distinctly involved in *β*-catenin association with E-cadherin and *α*-catenin. Accordingly, in IEC18 intestinal epithelial cells, overexpression of K-Ras led to *β*-catenin phosphorylation at tyrosines 142 and 654, inducing the dissociation from both E-cadherin and *α*-catenin [135]. Fer and Fyn promoted the phosphorylation of a GST-*β*-catenin construct at tyrosine 142, leading to the dissociation of *β*-catenin from *α*-catenin but not from E-cadherin. Y142F *β*-catenin mutant was resistant to Fer-induced loss of *α*-catenin binding. In contrast, Src and Yes were able to phosphorylate *β*-catenin at sites other than tyrosine 142 [135]. In mouse hearts, VEGF induced FAK activation, binding to VE-cadherin, and phosphorylation of *β*-catenin at tyrosine Y142, promoting the dissociation of *β*-catenin from VE-cadherin [136].

Even though phosphorylation-induced loss of catenin binding is an attractive mechanism to explain the reduction of cell-cell adhesion, accumulated evidence clearly shows that AJ phosphorylation cannot be directly linked to AJ disassembly in every case. Instead, the net effect is the overall sum of multiple actions to either increase or decrease AJ protein association, depending on the kinase involved and the specific tyrosines phosphorylated. For example, in 3Y1 fibroblasts [25] and MDCK cells [27] transformed with v-Src, E-cadherin was able to coimmunoprecipitate with *α*-catenin or *β*-catenin, respectively, even when v-Src induced a marked increase in E-cadherin phosphorylation. Further, v-Src activation reduced cell adhesion strength in L fibroblasts expressing an E-cadherin-*α*-catenin fusion construct that did not bind *β*-catenin, demonstrating that Src can inhibit cell adhesion independently of junction disassembly through *β*-catenin phosphorylation [30]. Keratinocytes induced to differentiate by culturing in high Ca^2+^ media displayed increased phosphorylation of *β*-catenin and *γ*-catenin, which correlated with an increased association of *α*-catenin and p120 with E-cadherin [40]. Further, p120 and *β*-catenin may be regulated independently. For example, Ras-induced transformation of MCF10A human mammary epithelial cells promoted the tyrosine phosphorylation of AJ components and a loss of E-cadherin/*β*-catenin binding concurrently with an increase in E-cadherin/p120 association [29]. Similar observations were made in IEC cells expressing K-Ras [135]. Consistently,* in vitro* phosphorylation and binding assays demonstrated that Src can directly phosphorylate p120 and *β*-catenin with markedly different outcomes: while *β*-catenin phosphorylation at Y654 reduced *β*-catenin affinity for an E-cadherin cytosolic domain, p120 phosphorylation increased E-cadherin binding [137]. In E8 chicken retina cells, p120-associated Fer was essential to maintain *β*-catenin binding to N-cadherin through phosphorylation and activation of the phosphatase PTP1B, which in turn was responsible for dephosphorylating *β*-catenin at tyrosine 654 [46]. *γ*-catenin phosphorylation can also lead to different results, depending on the kinase involved and the phosphorylated tyrosine. While Src-mediated phosphorylation at tyrosine 683 reduced the association of *γ*-catenin with E-cadherin and *α*-catenin, Fer-induced phosphorylation at tyrosine 549 increased *γ*-catenin binding to *α*-catenin [138].

Tyrosine phosphorylation has also been linked to AJ dissociation in the endothelium. Tyrosines 658 and 731 in the VE-cadherin tail are required for binding to catenins [139], since phosphomimetic mutations Y658E and Y731E in VE-cadherin constructs expressed in CHO cells prevented the binding to p120 and *β*-catenin, respectively. Expression of the same mutants prevented the formation of a tight barrier in these cells [139]. Similarly, Y658F VE-cadherin, but not wt or Y658E VE-cadherin, was able to bind to p120 in rat fat pad endothelial cells that lacked endogenous VE-cadherin [140]. Consistently, VEGF treatment in human pulmonary microvascular cells induced the loss of *β*-catenin and p120 binding to VE-cadherin, which correlated with phosphorylation of both VE-cadherin and *β*-catenin at tyrosine 654 [141], and expression of an Y658F/Y731F VE-cadherin mutant blocked VEGF-induced permeability and loss of VE-cadherin binding to *β*-catenin and p120 [141]. Further, overexpression of a catalytically inactive C459S SHP2 mutant in rat lung microvascular endothelial cells resulted in increased phosphorylation of VE-cadherin, p120, and *β*-catenin and reduced p120 association with VE-cadherin that was associated with a loss of barrier function [142]. VE-cadherin phosphorylation, however, does not always correlate with a decreased association with p120 or *β*-catenin. Early on, Esser et al. [74] showed that, in HUVECs, VEGF stimulation promoted VE-cadherin and catenin phosphorylation, but this treatment did not affect the level of cadherin/catenin coimmunoprecipitation, clearly dissociating the phosphorylation events from a loss of cadherin association with catenins. Similarly, histamine-induced VE-cadherin phosphorylation in HMEC-1 cells was not followed by a loss of VE-cadherin association with p120, *β*-catenin, or *γ*-catenin [143]. Moreover, while bradykinin treatment in HUVECs promoted VE-cadherin phosphorylation at tyrosine 658 and this phosphorylation was required for VE-cadherin endocytosis, internalized VE-cadherin was still associated with p120 [90]. Thus, multiple factors can promote AJ tyrosine phosphorylation without promoting a loss of cadherin binding. To directly assess the ability of Src-induced AJ phosphorylation to disassemble the adherens junction complex, increased tyrosine signaling in human dermal microvascular cells was induced by overexpression of a constitutively active (Y530A) Src construct or by inhibiting Csk activity [94]. Inhibition of Csk was achieved by overexpression of a kinase dead (K222R) mutant Csk that acts as a dominant negative [144]. While cells displayed markedly increased tyrosine phosphorylation, including VE-cadherin, the ability of endogenous VE-cadherin to colocalize and to coimmunoprecipitate with p120, *β*-catenin, and *γ*-catenin was not affected [94]. Y530A Src (but not DN-Csk) induced a dramatic loss of barrier function, as measured by monolayer gap formation, TEER, and albumin permeability [94], demonstrating that diminished cadherin/catenin association may not be required for endothelial barrier function loss.

Thrombin, another potent agent that induces a rapid increase in endothelial permeability, promoted the tyrosine phosphorylation of *β*-catenin without affecting its association with VE-cadherin, as measured by coimmunoprecipitation [145]. In HPAE cells, thrombin-induced monolayer gaps contained thin membrane projections that still connected the two adjacent cells [146]. Interestingly, the authors observed a reduction in the levels of colocalization of cadherin and catenins only in these projections, without a general loss of cadherin/catenins coimmunoprecipitation or colocalization in the rest of the cell body [146], suggesting that AJ disruption may occur only at the sites of adhesion loss. These projections are morphologically identical to the “finger-like” structures observed after TGF-*β* treatment in bovine pulmonary artery cells [147], the discontinuous junctions induced by TNF-*α* [148], and the focal adherens junctions shown by Huveneers et al. to contain vinculin molecules linking VE-cadherin to radial actin junctions [149]. In these other studies, VE-cadherin and catenins remained present within these thin structures after the formation of the gap and are probably responsible for maintaining the connection between the two endothelial cells surrounding the gap [147–149]. In fact, at least in the case of TGF-*β*, it appears that gap formation precedes a loss of catenin staining [147], suggesting the possibility that adherens junction complex disruption is a consequence, and not a cause, of the sustained loss of adhesion. Focal adherens junctions may be involved in junctional formation and remodeling* in vitro* [149, 150] and this remaining connection might be critical* in vivo* to ensure a fast gap closure as proposed by Baluk et al. [151] after detailed description of substance P-induced gap formation in rat trachea venules. Detailed experiments performing multicolor live imaging at high resolution will be required to set this issue.

The regulation of the adherens junction complex may also involve phosphorylation of serine and threonine. In HPAE cells, thrombin effects were associated with a PKC-dependent dephosphorylation of VE-cadherin and *β*-catenin and p120 phosphorylation [146]. As VE-cadherin phosphorylation was assessed by 2D electrophoresis rather than phosphotyrosine blots, it is possible that many of the observed dephosphorylation events occurred in serine and threonine residues, rather than tyrosines [146]. In fact, PKC*α* was shown to mediate thrombin- and LPS-induced p120 phosphorylation at serine 879, leading to the dissociation from VE-cadherin and AJ disassembly [152]. E-cadherin serine phosphorylation regulates *α*-catenin, *β*-catenin, and *γ*-catenin binding, and mutation of a cluster of eight serine residues from S838 to S853 prevented E-cadherin binding to the catenins (as measured by E-cadherin IP of ^35^S-labeled cells) and abolished the ability of E-cadherin to promote cell aggregation, a method to determine cell-cell contact strength [153]. Structural studies demonstrated an increased affinity between phosphorylated E-cadherin and *β*-catenin [154, 155]. E-cadherin serine phosphorylation may be mediated by casein kinases (CK). CK-II-mediated E-cadherin serine phosphorylation increased *β*-catenin binding in NIH3T3 expressing exogenous mouse E-cadherin [156, 157].* In vitro*, CK-II phosphorylated wild-type E-cadherin, while S840A, S853A, and S855A E-cadherin mutants were resistant to CK-II-mediated phosphorylation [156]. In another study, CK-I phosphorylated E-cadherin at S846* in vitro* [158], while CK-II was able to phosphorylate S846A E-cadherin mutant, but not an E-cadherin construct in which serines 849, 852, and 855 were mutated to alanine, suggesting that CK-I and CK-II phosphorylate E-cadherin at close but different sites. In GST pull-down assays, S846D phosphomimetic mutant showed decreased binding to *β*-catenin but did not modify the association with p120 [158]. Serines 846, 849, 852, and 855 in E-cadherin correspond to S742, S745, T748, and S751 in VE-cadherin, but it is unknown whether casein kinases can phosphorylate VE-cadherin. Suggestively, all phosphorylatable residues are conserved in that region, with the only exception of a serine for threonine substitution and two reciprocal substitutions for acidic amino acids (S_852_S_853_E_854_ → T_748_D_749_S_750_), arguing to support a conserved need for negative charges in this domain. CK-I is best known as a component of the *β*-catenin destruction complex that is part of the Wnt pathway [159], but it was also found to phosphorylate p120 and *α*-catenin. In response to Wnt3a, CK-I promoted p120 phosphorylation, linking p120 to Wnt-mediated *β*-catenin transcription [160]. In response to EGF, CK-II phosphorylated *α*-catenin at serine 641, leading to the release of *β*-catenin and an increase in *β*-catenin transactivation [161]. Recently, it was also shown that both CK-I and CK-II can phosphorylate *α*-catenin* in vitro* on a cluster of serine and threonine residues at the *α*-catenin flexible linker [162], including S641. Expression of nonphosphorylatable and phosphomimetic *α*-catenin mutants in MDCK cells in a model of monolayer fragmentation suggested that phosphorylation of *α*-catenin at this region promotes cell-cell adhesion strength and monolayer integrity. This effect, however, was not due to changes in junctional assembly, as all mutants associated with E-cadherin or *β*-catenin to the same extent [162].

Overall, the biochemical data suggest that the observation of a particular phosphorylation event may not necessarily imply that large changes in cadherin/catenin association will be detected. While this fact does not negate an important role in junctional phosphorylation in the regulation of the adherens junction assembly and disassembly, several conclusions can be drawn from all the accumulated evidence. First, tyrosine signaling must act in concert with other pathways to promote junctional disassembly and loss of cell adhesion, as direct kinase activation does not always lead to junctional loss. Second, kinase deficient models and small molecule inhibitors demonstrated that a basal level of tyrosine phosphorylation is required for junctional maintenance and that a precise regulation of the levels of activity may be needed in order to ensure normal cell adhesion. Third, fluorescence imaging showed that the loss of cell-cell contacts is reversible and geographically limited, and thus biochemical methods may lack the sensitivity to detect small but important changes. It can be envisioned that junctional disassembly and loss of cadherin/catenin association may be strictly limited to subcellular regions immediately surrounding the formation of a gap or transmigrating leukocyte and temporally restricted to the initiation of such event, reforming quickly to allow junctional recovery.

## 7. FAK Has Dual Roles

Although most of the efforts to understand tyrosine kinase signaling in the regulation of endothelial contacts have been focused on SFKs, an important role emerged for the focal adhesion kinase family of tyrosine kinases. As its name implies, FAK is best known in the context of integrin signaling (reviewed in [163]). Notably, FAK and Src have complex interactions. FAK is Src substrate, but it can also mediate Src activation, placing FAK both upstream and downstream of Src [163]. FAK has multiple roles regulating epithelial junctions in collective cell migration and metastasis, which are beyond the scope of this review [164, 165]. As with other tyrosine kinase signaling in endothelial cells, FAK has been described to act as a promoter of AJ formation and strengthening as well as an inducer of AJ disassembly. In rat lung microvascular endothelial cells, FAK mediated the increase in TEER induced by hyperosmolarity [166] as well as the recovery of the barrier function after a transient loss stimulated by hydrogen peroxide treatment [167]. Similarly, the recovery of HPAECs from thrombin-induced loss of barrier function was dependent on FAK activity [168]. In that study, expression of FAK related nonkinase (FRNK, which blocks endogenous FAK activity [163]) decreased basal TEER and prevented the recovery after thrombin. FRNK blocked p190 RhoGAP phosphorylation and promoted more sustained Rho activation. Further, cationic liposome-mediated FRNK expression to lungs increased fluid permeability in perfused isolated mouse lungs, demonstrating a role for FAK activity in whole tissues [168]. FAK activation downstream of the PAR1 thrombin receptor was mediated by G*β*1 association with Fyn, leading to the association of activated FAK with p120 [169]. FAK was also recruited to the AJ in cells treated with sphingosine 1-phosphate (S1P), an agonist that promotes endothelial barrier strengthening [170]. In these experiments, FAK coprecipitated with *β*-catenin after S1P treatment, and knockdown of *β*-catenin prevented the association between FAK and VE-cadherin. Moreover, Lyn kinase, an SFK, promoted the stabilization of endothelial barrier through phosphorylation of FAK at tyrosines 576/577 and 925 [171] and FAK-deficient mouse endothelial cells displayed increased permeability to FITC-dextran [172]. Thus, there are many strong indications* in vitro* and* ex vivo* that FAK promotes junctional assembly and endothelial barrier function. Nevertheless, published data also argues in favor of another role in promoting barrier disruption. FAK knockdown in immortalized human microvascular cells increased basal TEER [173] and FRNK expression blocked the VEGF-induced increase in permeability in isolated porcine coronary venules and HUVECs [174]. Further, as opposed to the observations made in mouse lungs [168], direct transfection of FRNK into pig coronary venules did not affect basal permeability but prevented neutrophil-induced leakage [175]. Confirming a role for this kinase in VEGF effects, VEGF-induced permeability was abrogated by the FAK inhibitor PF-562271 in HPAECs and by expression of a kinase dead FAK in mouse endothelial cells [136]. Furthermore, FAK can directly phosphorylate* in vitro* VE-cadherin at Y658 [176].

A definitive proof for a requirement of endothelial FAK promoting endothelial barrier function* in vivo* was provided by Schmidt et al. [177], by showing that conditional FAK deletion in the endothelium promoted features of acute lung injury, such as hemorrhage, edema, and neutrophil accumulation. The authors attributed the phenotype to increased activation of RhoA mediated by p115RhoGEF. However, Chen et al. [136] showed that VEGF-induced permeability was abrogated in mice expressing a kinase dead (K545R) FAK in the endothelium. The authors used a mouse model in which one floxed FAK allele was deleted in the endothelium by tamoxifen, while the other allele consisted in either wild-type FAK or a K545R FAK knock-in, thus rendering the mouse ECs with either active or inactive FAK, respectively. Using this model, they demonstrated that FAK is required for VEGF-induced vascular leakage in the dermis. This effect was mimicked by the FAK inhibitor PF-562271 [136]. K545R FAK knock-in mice also showed diminished VEGF-mediated tumor cell extravasation and VE-cadherin Y658 phosphorylation [176]. The data presented above clearly implicates at least two distinct roles for FAK, one as a kinase that is required for normal junctional assembly and another role downstream of the edemagenic effects of VEGF. A possible explanation for these seemingly contradictory roles may lay in FAK's ability to counteract Rho activation (which may be dominant in junctional assembly and in response to thrombin) and to mediate Src activation (a critical step in VEGF signaling). Other Cre-inducible FAK knock-in mice have been developed, including the nonphosphorylatable mutants Y397F and Y861F and the phosphomimetic Y397E [178]. It will important to determine the similarities and differences between the phenotypes of these point mutants versus the FAK null and the kinase dead mutant described above, as they may provide new insight to understand the differential roles of FAK in cell adhesion.

## 8. The Other Side of the Coin: Tyrosine Phosphatases

Adherens junction proteins can bind to at least 12 distinct tyrosine phosphatases (reviewed in [15, 179]). Of those, several have been shown to affect intercellular adhesion strength, either by directly dephosphorylating adherens junction components or by indirectly affecting their phosphorylation levels through the modulation of RTK signaling and SFK activation. A discussion of the most relevant findings relating to endothelial barrier regulation is provided below.

### 8.1. SHP2

Src homology-2 (SH2) domain-containing phosphatase 2 (SHP2, also called PTP11, PTP-1D, or PTP-2C) is a ubiquitously expressed phosphatase that is associated with multiple neoplastic malignancies, as well as three closely related inherited developmental disorders [180–182], the Noonan syndrome, the Noonan-like disorder with multiple giant cell lesion syndrome, and the LEOPARD syndrome, that include, among many other defects, lymphatic malformations and bleeding difficulties [183].

A first indication that SHP2 may regulate endothelial cell-cell contacts was provided by Ukropec et al. [184]. In HUVECs, thrombin promoted a transient increase lasting less than 30 minutes of several phosphotyrosine bands in VE-cadherin immunoprecipitates that comigrated with p120, *β*-catenin, and *γ*-catenin. This increase correlated with SHP2 phosphorylation and a loss of SHP2 in the VE-cadherin immunoprecipitates. Direct association of SHP2 with *β*-catenin was likely, since a far-Western blot assay demonstrated that a construct consisting of GST-tandem SHP2 SH2 domains bound to isolated *β*-catenin, but not VE-cadherin, p120, or *γ*-catenin from HUVEC lysates [184]. Consistently, Timmerman et al. showed that thrombin promoted transient *β*-catenin phosphorylation that lasted 15–30 minutes [145]. Thrombin treatment induced Src activation and SHP2 phosphorylation at Y542 with kinetics that correlated with the loss of *β*-catenin phosphorylation and the recovery of TEER. To prove that SHP2 mediated the recovery, it was shown not only that SHP2 immunoprecipitated from cells treated with thrombin was able to dephosphorylate *β*-catenin* in vitro*, but also that SHP2 knockdown prolonged *β*-catenin phosphorylation and thrombin-induced TEER loss [145]. Additionally, SHP2-mediated regulation of cell adhesion may involve Rho GTPases, key mediators of the thrombin response. Early on, it was shown that SHP2 inhibition in fibroblasts promoted Rho activation [185]. In vascular smooth muscle cells, SHP2 mediated the angiotensin II-induced dephosphorylation and inactivation of p190RhoGAP, leading to increased RhoA activation [186]. Proof that this pathway was active in endothelial cells was provided in PAECs [142]. In these cells, inhibition of SHP2 activity by expression of the inactive mutant C459S SHP2 or treatment with the pharmacological SHP2 inhibitor NSC-87877 reduced p190RhoGAP activity and promoted RhoA activation as measured by GST-RBD pull-down assays. SHP2 inhibition reduced basal monolayer resistance in PAECs and promoted an increase in phosphorylated VE-cadherin and *β*-catenin as measured by IP and phosphotyrosine Western blot [142]. More recently, it was shown that both LPS and thrombin treatment induced a reduction in lung SHP2 activity and association with FAK [187]. Further, liposomal delivery of a constitutively active (D61A) SHP2 mutant reduced pulmonary edema in mice challenged with LPS or* Pseudomonas aeruginosa* [187], suggesting that SHP2 plays an important role in preventing acute lung injury.

SHP2 also regulates the response to other vasoactive mediators. An early observation that VEGFR2 phosphorylation in response to VEGF-165 was much higher in HUVECs grown on vitronectin than in cells grown on collagen I [188] was attributed to differential involvement of SHP2 through direct association with phosphorylated VEGFR2 [189]. SHP2 may indirectly promote Src activation by dephosphorylating the Csk regulator Cbp and inactivating Csk [190]. In fact, SHP2 mediated Src and PI3K activation after VEGF treatment by inducing the dissociation of Csk from VE-cadherin in BAECs [65]. SHP2 interactions with Gab1, an adaptor protein that strongly associates with both SHP2 and PI3K [191, 192], may also explain in part why SHP2 is required for VEGF-induced PI3K activation. In porcine aortic endothelial cells, flow induced the formation of a complex involving SHP2, Gab1, and PI3K that was required for flow-induced eNOS phosphorylation [193]. Flow also induced SHP2 and Gab1 translocation to the plasma membrane in BAECs [194], as well as increased SHP2 and PECAM-1 association in both BAECs [194] and HUVECs [195]. A complex involving SHP2, Gab1, and Grb2 also mediated PI3K activation downstream of FGFR1 receptors [196, 197]. Importantly, FGF2, a ligand of FGFR1, promoted the formation of tight capillaries in a mouse corneal angiogenesis model [198]. SHP2 mediated regulation of adherens junction stability downstream of FGF appears to involve a different mechanism. In BAECs, inhibition of FGF signaling promoted VE-cadherin internalization and dissociation from p120, an effect with important consequences* in vivo*, since inhibition of FGF signaling in rats using adenoviral delivery of FGF traps destabilized the vasculature integrity and promoted vascular barrier loss [199]. Subsequent research from the same group showed that overexpression of a dominant negative form of FGFR1 (FGFR1DN) reduced VE-cadherin association with SHP2 and p120 [200]. FGFR1DN induced the phosphorylation of VE-cadherin, but not p120, as well as a loss of junctional localization of a VE-cadherin-GFP construct. Phosphorylation of VE-cadherin at Y658 was required for the loss of junctional localization, as Y658F VE-cadherin-GFP construct was resistant to FGFR1DN-induced junctional loss. Confirming a causal role for the loss of SHP2 in this model, overexpression of SHP2 partially rescued FGFR1DN-induced loss of TEER [200].

### 8.2. Dep1

Dep1 (also called CD148 and PTP*η*) is a ubiquitously expressed phosphatase that was originally cloned from a HeLa cDNA library [201].* In vitro*, Dep1 was found to bind directly to Src [202] and to dephosphorylate ZO-1, occludin [203], p120, and *β*-catenin [204]. Interestingly, Dep1 expression increased with cell density in WI38 and AG1518 fibroblasts [201]. Expression of Dep1 in transformed rat thyroid PCMPSV cells increased Src activity via Y527 dephosphorylation (corresponding to Y530 in human Src) without affecting the level of phosphorylated Y416 (Y419 in human Src). This led to an increase in the tyrosine phosphorylation of FAK and paxillin and overall increased adhesion to the substratum [202]. Experiments using GST pull-downs showed that Dep1 can interact with phosphorylated junctional proteins in endothelial [205] and epithelial [203] cells. A substrate trapping (D/A mutant) Dep1 catalytic domain coprecipitated with p120, *β*-catenin, and *γ*-catenin in lysates from HUVECs treated with pervanadate, but not control cells [205]. Similar to observations in HUVECs, GST-C/S Dep1 bound to ZO1, occludin, Src, and p120 in lysates from MCF10A mammary epithelial cells pretreated with pervanadate, but not from control cell lysates [203]. Expression of Dep1 in MDCK-II cells promoted monolayer barrier function, as measured by increased TEER and reduced FITC-dextran permeability after a calcium switch assay [203]. The role of Dep1 was also studied in A431D epidermoid cervical carcinoma cells (that lack endogenous classical cadherins [206]), in which E-cadherin was reexpressed [204]. Coexpression of Dep1 promoted an increase in junctional E-cadherin in these cells, which was dependent on E-cadherin/p120 association, since Dep1 was unable to increase junctional association of 764EED → AAA E-cadherin [204], a mutant that is unable to bind p120 [121]. In a calcium switch assay, wild-type but not C/S Dep1 potentiated adhesion-mediated Rac activation without affecting CDC42 or Rho GTP levels. Rac activation by Dep1 was also dependent on the association between p120 and E-cadherin, as it was observed in cells expressing wild-type but not 764AAA E-cadherin [204]. Even though* in vitro* Dep1 was shown to dephosphorylate p120, in A431D cells Dep1 promoted an increase in the phosphorylation level of Y228 p120 after calcium addition. Importantly, the authors found that Dep1 expression also increased junctional VE-cadherin when expressed in HUVECs, although it remains unexplored whether the same p120-dependent mechanism is governing the action in HUVECs [204].

Dep1 was also shown to be expressed in the endothelium* in vivo* and to colocalize with VE-cadherin [207], but its role is not completely understood. Homozygous expression of a mutant Dep1 that lacks the phosphatase domain is embryonically lethal. Embryos die at E11.5 from multiple vascular defects including enlarged vessels and increased endothelial proliferation [208]. Paradoxically, mice completely lacking Dep1 were viable and fertile [209], suggesting that the phenotype of knock-in expressing Dep1 mutant may be due to a dominant function of this construct. However, Dep1 knockout mice, while viable, displayed deficient cerebral arteriogenesis in a model of left common carotid artery occlusion [210], thus confirming a role for wild-type Dep1 in the vasculature* in vivo*. A mechanism for the Dep1-mediated regulation of cell proliferation via the modulation of VEGFR2 signaling was proposed by Lampugnani et al. [60]. Endothelial cell contact inhibition of proliferation correlated with reduced VEGF signaling in confluent cells. The inhibition of VEGF signaling was dependent on Dep1 activity, together with the expression of *β*-catenin and its interaction with VE-cadherin. Expression of a catalytically inactive C/S Dep1 mutant or siRNA-mediated Dep1 knockdown restored VEGF-induced VEGFR2 phosphorylation and cell proliferation in confluent cells [60], suggesting a negative role of Dep1 in VEGF signaling mediating contact inhibition. This phosphatase, however, may have opposite effects on different VEGFR2 downstream signals. In HUVECs, Dep1 knockdown potentiated VEGF-induced VEGR2 phosphorylation at multiple tyrosine residues, as well as the phosphorylation of PLC*γ*, eNOS, and Erk1/2, but prevented VEGF-induced Akt activation [211]. Consistent with the previously described role for Dep1 in Src activation [202], this was associated with reduced VEGF-induced Src activation in Dep1 knockdown cells due to increased Src phosphorylation at Y530 and reduced association between Src and Gab1 [211]. In stark contrast, morpholinos directed at either one of the two zebrafish Dep1 genes (Dep1a or Dep1b) promoted vascular defects that could be rescued by PI3K inhibition, suggesting that Dep1 in zebrafish negatively regulates PI3K [212]. Reciprocally, Dep1 can be phosphorylated by Src and Fyn on Y1311 and Y1320, leading to the dephosphorylation of Y530 Src by Dep1 [213]. BAECs expressing Y1311F/Y1320F Dep1 mutant did not display Src dephosphorylation at Y530 after VEGF, while Erk activation was similar in wild-type and YYFF Dep1-expressing cells. Dep1 knockdown or the YYFF mutant in HUVECs prevented the VEGF-induced VE-cadherin phosphorylation at S665, monolayer gap formation, and increase in FITC-dextran permeability [213].

### 8.3. VE-PTP

VE-PTP (also called R-PTP-*β*) is an endothelial-specific transmembrane tyrosine phosphatase that was cloned from a bEnd5 cDNA library [214]. The first demonstration that VE-PTP interacts with VE-cadherin was provided by Nawroth et al. by showing that exogenously expressed VE-PTP and VE-cadherin coimmunoprecipitated from COS-7 lysates [215]. In COS-7 cells expressing VE-PTP, VE-cadherin, and VEGFR2, VE-PTP was able to dephosphorylate VE-cadherin [215]. VE-PTP may have an important role in promoting junctional assembly and in maintaining cell adhesion. VE-PTP relocalization to cell-cell contacts from the endosome recycling compartment and association with VE-cadherin increased with endothelial confluence in bEnd3 cells and HUVECs, suggesting a role in AJ maturation [119]. Further supporting this notion, VE-PTP was able to bind and dephosphorylate *γ*-catenin [119], a junctional component that also increased binding to VE-cadherin with cell confluence [72]. Similarly, expression of VE-PTP in CHO cells increased VE-cadherin association with *γ*-catenin, suggesting that this phosphatase can enhance VE-cadherin/*γ*-catenin binding, but a mutant VE-cadherin in which all C-terminal tyrosine residues were replaced by phenylalanine also bound *γ*-catenin in the presence of VE-PTP, demonstrating that the effect of VE-PTP in *γ*-catenin binding was independent of the tyrosine phosphorylation level of VE-cadherin [119]. Conversely, VE-PTP knockdown reduced VE-cadherin-mediated adhesion, increased endothelial permeability to FITC-dextran, and enhanced neutrophil transmigration. Further, neutrophil or T-cell attachment to bEnd5 cells induced the dissociation of VE-PTP from VE-cadherin and promoted VE-cadherin, *β*-catenin, and *γ*-catenin phosphorylation [119]. Leukocyte-induced VE-PTP dissociation from VE-cadherin was found to be mediated by VCAM-1 through a pathway that involved Rac1, ROS generation, and Pyk2 activation. Blocking antibodies to VCAM-1 prevented T-cell-induced dissociation, while direct VCAM-1 cross-linking promoted VE-PTP/VE-cadherin dissociation [120]. LPS and VEGF also promoted the dissociation between VE-PTP and VE-cadherin* in vivo* [216]. To determine the role of this dissociation, the authors generated knock-in mice harboring two fusion proteins, VE-cadherin-FKBP and VE-PTP-FRB. When treated with the small molecule rapalog, these VE-cadherin and VE-PTP chimeras were locked in a heterodimeric conformation, thus preventing the dissociation induced by VEGF or leukocyte attachment. Consistent with a role for VE-PTP/VE-cadherin dissociation in TEM, rapalog injections in these knock-in mice prevented leukocyte extravasation in the IL-1*β*-induced inflammation of the cremaster muscle model, without affecting leukocyte attachment or rolling, and reduced LPS-induced increase of PMN in bronchoalveolar lavage (BAL) fluid. Rapalog injections also diminished VEGF and LPS-induced vascular leakage (measured by a Miles assay and protein content in BAL fluid, resp.) [216]. A role for VE-PTP in vascular permeability was also found in a zebrafish model, suggesting that VE-PTP roles are highly evolutionarily conserved [217]. In this model, injection of morpholinos against zebrafish VE-PTP causes blood cell aggregates, hemorrhage, and hyperpermeability to tetramethylrhodamine-dextran. Electron microscopy demonstrated that, in VE-PTP morphants, 60% of tail vessel ECs did not have junctional complexes, supporting the notion that VE-PTP maintains zebrafish VE-cadherin adhesions [217].

VE-PTP also plays a critical role in vascular development via its regulation of Tie2 and VEGFR2 signaling. VE-PTP null mice [218] or mice expressing a truncated form of VE-PTP [219] are not viable and embryos die at E8.5-10 due to vascular malformations. Allantois explants from VE-PTP-mutant mice displayed enlarged vessels with endothelial cells growing in sheets [219]. Moreover, antibodies against VE-PTP induced vessel enlargement in allantois explants that resemble observations in VE-PTP-mutant mice [220]. These antibodies did not induce vessel enlargement in Tie2^−/−^ allantois, demonstrating that Tie2 mediated this effect. Further supporting VE-PTP-Tie2 axis controlling vessel growth, daily injections of anti-VE-PTP antibodies for 7 days in young mice induced vessel enlargement in the tongue and Tie2 phosphorylation in lung lysates [220]. VE-PTP was shown to associate with Tie2 in bEnd5 cells and to dephosphorylate exogenously expressed Tie2, but not VEGFR2, in COS-7 [214]. VE-PTP and Tie2 may act as a negative regulator of VEGF receptor activation and downstream signaling. VE-PTP knockdown prevented VEGF-induced tube formation in telomerase-immortalized human microvascular endothelial cells grown on a 3D collagen I matrix [221]. This was associated with an increase in VEGF-induced phosphorylation of VEGFR2 and proliferation, without any effect in apoptosis [221]. Further, VE-PTP-deficient embryoid bodies displayed increased angiogenic sprouting and Y1175 VEGFR2 phosphorylation [222]. The same study found that VE-PTP in stalk cells dephosphorylated VEGFR2, in a mechanism that required Tie2. Similarly, in porcine aortic endothelial cells lacking Tie2, VE-PTP did not coprecipitate with VEGFR2, even though in an* in vitro* assay VE-PTP was able to directly dephosphorylate VEGFR2 [222]. The mechanism downstream of VE-PTP may also involve the regulation of VE-cadherin phosphorylation, as angiogenic sprouts in VE-PTP knockout embryonic bodies showed increased pY658 VE-cadherin after VEGF treatment [222]. This effect might be specific to VEGF-induced phosphorylation, since in bEnd5 cells VE-PTP inhibition via blocking antibodies or siRNA-mediated knockdown induced Tie2 phosphorylation without promoting an increase in VE-cadherin phosphorylation [220].

### 8.4. PTP*μ*

PTP*μ* is a transmembrane receptor protein tyrosine phosphatase that was isolated using degenerated PCR primers from mouse brain cDNA based on its homology to other tyrosine phosphatases [223]. It contains an immunoglobulin domain and four fibronectin type III repeats and can mediate homophilic interactions through its extracellular domains [224]. PTP*μ* localizes to cell-cell junctions in MvLu mink lung epithelial cells and coprecipitates with E-cadherin, *β*-catenin, and *α*-catenin [225].* In vitro*, PTP*μ* was able to bind directly to the intracellular domain of E-cadherin but not to *α*-catenin or *β*-catenin. Pervanadate treatment did not prevent the coprecipitation of PTP*μ* with E-cadherin, suggesting that PTP*μ* can also associate with hyperphosphorylated cadherins [225]. However, a temperature-sensitive v-Src construct promoted E-cadherin phosphorylation and dissociation of PTP*μ* in WC5 neonatal rat cerebellar cells [226], suggesting that tyrosine phosphorylation events can regulate E-cadherin/PTP*μ* association. The PTP*μ* binding site in E-cadherin was located to the C-terminal 38 amino acids, close to the *β*-catenin binding site [226]. Interestingly, PTP*μ* can sustain E-cadherin adhesion in LNCaP prostate carcinoma cells through a mechanism that involves scaffolding, but not catalytic activity [227]. AJ/PTP*μ* association is not restricted to E-cadherin, as PTP*μ* coprecipitated with E-cadherin, N-cadherin, and R-cadherin in rat lung extracts [226] and promoted neurite outgrowth of chicken retinal ganglion cells grown on N-cadherin-coated surfaces [228]. In human lung microvascular ECs, PTP*μ* coprecipitated with VE-cadherin, and* in vitro* GST pull-downs demonstrated a direct interaction between PTP*μ* and VE-cadherin [229]. This interaction may be important in regulating endothelial barrier, because PTP*μ* knockdown or expression of catalytically inactive constructs increased permeability to albumin. This effect may be due to regulation of VE-cadherin phosphorylation, as PTP*μ* overexpression in immortalized HMEC-1 cells reduced basal VE-cadherin tyrosine phosphorylation [229]. Expression of PTP*μ* in endothelial cells appears to be variable. PTP*μ* expression increased severalfold with increasing monolayer confluence in HUVECs [230] and bovine aortic endothelial cells [231].* In vivo*, its expression may be restricted to arteries. Immunofluorescence studies showed that PTP*μ* expression was higher in arterioles and arteries than in veins of multiple rat tissues [231], and PTP*μ*-LacZ knock-in mice displayed *β*-galactosidase activity in arterioles and capillaries, but not in veins or in fenestrated endothelium [232]. This observation could explain at least in part why venules display increased Src activation and VE-cadherin phosphorylation [90]. However, the fact that pervanadate (which blocks the activity of multiple phosphatases, including PTP*μ* [233]) increased VE-cadherin phosphorylation only in venules in the cremaster vasculature [91] argues against PTP*μ* differential expression as the sole explanation.

### 8.5. PTP1B

PTP1B is a ubiquitously expressed nonreceptor tyrosine phosphatase that holds the record of being the first tyrosine phosphatase purified and characterized [234–237]. PTP1B can dephosphorylate multiple phosphotyrosine-containing proteins, including several receptor and receptor-associated tyrosine kinases [238], and as such it plays a critical role in heart disease, insulin resistance, and leptin regulation, as well as in multiple neoplastic disorders [238]. The ability of PTP1B to associate with the adherens junctions was first shown by Balsamo et al. [239, 240] in chicken retina cells. In those cells, N-cadherin copurified with tyrosine phosphorylated PTP1B and with nonphosphorylated *β*-catenin. N-cadherin binding to PTP1B requires PTP1B tyrosine 152 phosphorylation [240, 241]. In turn, PTP1B leads to *β*-catenin dephosphorylation [46, 239]. Similar to PTP*μ* [226], PTP1B/N-cadherin association is mediated by a region near the C-terminus of N-cadherin, close to the *β*-catenin binding site, although *β*-catenin and PTP1B do not appear to compete for N-cadherin binding [242]. Besides a possible direct role in dephosphorylating *β*-catenin, it is possible that PTP1B may affect junctional stability through the direct modulation of Src activity [243–246]. For example, fibrinogen binding to *α*_IIb_ *β*_3_-integrin in platelets triggers PTP1B recruitment to a complex involving Src, Csk, and integrins, which leads to Csk dissociation and Src activation through dephosphorylation of tyrosine 530 [244]. PTP1B is recruited in Src-dependent fashion, because pretreatment with PP2 blocks PTP1B association with *β*_3_ integrin. Thus, not only can PTP1B be activated by tyrosine kinase signaling, but it can also promote tyrosine kinase activation. However, little is known about whether a PTP1B-Src axis is important in the endothelium. PTP1B was shown to bind and dephosphorylate VEGFR2* in vitro*, and expression of wild-type PTP1B, but not a C/S catalytically inactive mutant, prevented VEGF-induced VEGFR2 phosphorylation and Erk, but not p38, in HUVECs [247]. Conversely, PTP1B knockdown increased VEGF-induced VEGFR2 phosphorylation and Erk activation, without increasing basal VEGFR2 signaling. Consistent with an increase in VEGFR2 signaling, blocking PTP1B activity by expression of PTP1B C/S mutant or by PTP1B knockdown induced an increase in VE-cadherin tyrosine phosphorylation and a reduction in TEER [247]. PTP1B can coprecipitate with p120, *β*-catenin, and VE-cadherin in rat lung microvascular endothelial cells as well as mouse lungs [248]. LPS treatment in mice reduced the association between PTP1B and *β*-catenin. More importantly, expression of an oxidation-resistant PTP1B mutant reduced LPS-induced lung edema [248].

## 9. Concluding Remarks and Perspectives

**Figure 4:**
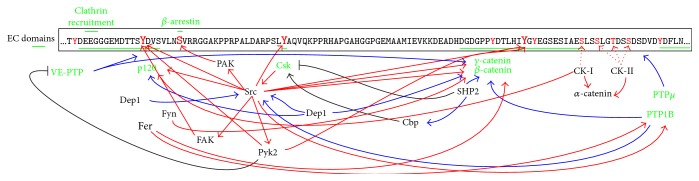
The net effect on cadherin tail phosphorylation depends on the action of multiple kinases and phosphatases. The VE-cadherin cytoplasmic region contains multiple phosphorylatable residues (in red) located in or near the JMD and CBD domains responsible for catenin binding (marked in green). Larger font highlights tyrosines 658, 685, and 731, together with serine 665, which have been more intensely studied. The overall phosphorylation status is the effect of a network of kinase (red arrows) and phosphatase (blue arrows) activities. These kinases and phosphatases can modify the cadherin tail and/or associated catenins directly or indirectly via the regulation of other associated kinases and phosphatases. Dotted arrowheads: CK-I and CK-II activity was shown to phosphorylate homologous residues in E-cadherin tail.

This year marks the 30th anniversary of the first detection of phosphotyrosine at the cell-cell junctions [20]. Since that initial discovery, intense research was aimed at understanding the mechanisms by which the adherens junction phosphorylation is regulated and at determining the functional effect of such phosphorylation events. It is now known beyond doubt that cadherin and catenin phosphorylation is a common event that occurs at multiple tyrosine residues, as a result of a complex balance of the multiple tyrosine kinases and phosphatases that interact with junctional proteins (Figure 4). While massive kinase activation or phosphatase inhibition leads to a dramatic loss of cell adhesion, there is an incomplete understanding of the details of the adherens junction regulation in cells with limited, regulated tyrosine kinase activation.

The ability of SFKs to phosphorylate VE-cadherin and the requirement for SFK activation downstream of multiple receptors, including VEGFR2 and ICAM-1, have been demonstrated. Nevertheless, it became clear that simply the observation of SFK activation cannot predict a loss of endothelial barrier function [94]. The findings that in mice SFKs may be active in the venular endothelium [90] and that VE-cadherin is phosphorylated in the absence of proinflammatory stimuli [88–91] prompt revisiting the role of VE-cadherin tyrosine phosphorylation in barrier function. One possibility is that SFK-mediated VE-cadherin phosphorylation may act as a gatekeeper to allow specific vascular beds to respond to proinflammatory agents that may trigger the loss of cell adhesion through the activation of other parallel signaling pathways. However, other mechanism(s) by which SFK signaling may crosstalk with other pathways cannot be excluded, such as the direct regulation of Rho GTPases as observed downstream of several tyrosine kinases and phosphatases [133, 142, 168, 177, 185, 204]. Moreover, loss of junctional VE-cadherin coincided* in vivo* with cadherin dephosphorylation of specific tyrosines [90, 91] and the inability to phosphorylate Y685 led to increased leakage in angiogenic tissues [92]. These observations raise the possibility that junctional components may require cycles of phosphorylation and dephosphorylation to enable the relocalization to different membrane compartments, as it is the case in focal adhesion turnover [249, 250]. In that scenario, dynamic changes in cadherin phosphorylation may allow transient binding to adaptor molecules. Endothelial gap formation can coincide with the generation of the so-called focal adherens junctions, which recruit vinculin to link cadherins and catenins to radial actin fibers [149], a process that may not be required for the formation of the gap itself, but for enabling the recovery of cell adhesion. However, it is not known whether vinculin association or any other scaffolding protein with the adherens junctions in this context requires a change in VE-cadherin and/or catenin phosphorylation.

A potential mechanism that stands out for its simplicity and logic is the regulation of the association of VE-cadherin to catenins by differential phosphorylation events. The well-known role of p120 in preventing VE-cadherin endocytosis [3, 125, 126, 128, 129] and the inability of a phosphomimetic Y658E VE-cadherin mutant to bind p120 [139, 140] support a model in which VE-cadherin phosphorylation drives the loss of p120 binding and thus endocytosis, leading to disruption of cell adhesion. However, most of the available biochemical data do not validate this model and strongly suggest that even with dramatic changes in VE-cadherin phosphorylation and/or loss of barrier function at least the majority of VE-cadherin remains bound to p120 [74, 90, 94, 143, 145, 146]. Should we disregard then this potential mechanism? We probably should not. The formation of endothelial gaps may require the loss of only a small subset of junctional VE-cadherin/p120 complexes, which would render the biochemical approaches not sensitive enough to detect small but important changes in junctional protein association. Alternatively, some stimuli may require loss of p120 binding and VE-cadherin endocytosis, while others may act via the dissociation of the bridge between *β*-catenin, *α*-catenin, and the actin cytoskeleton. Additionally, it is possible that some phosphorylation events in VE-cadherin may be a consequence, and not a cause, of catenin dissociation. The adherens junctions associate with multiple tyrosine kinases [46, 65, 66, 93, 135, 169, 172] and phosphatases [119, 145, 184, 201, 204, 205, 213], and both p120 and *β*-catenin can bind and recruit them to the junction or, alternatively, compete for a binding site and displace kinases and phosphatases from binding VE-cadherin, with the overall effect of modulating the levels of junctional phosphotyrosine (Figure 4). The exact temporal relationship in the set of events leading to the disruption of cell adhesion is still under study. Lastly, changes in tension at the junction due to catenin-regulated activity of Rho GTPases [251] not only may affect the ability of VE-cadherin to transduce mechanosensory stimuli [68] but could also potentially regulate RTK signaling and the levels of VE-cadherin phosphorylation and cell-cell adhesion. In fact, pharmacological inhibition of MLCK [147], ROCK [148, 149], or myosin II [149] prevented the formation of focal adherens junctions, demonstrating that actomyosin-mediated tension is critical for junctional remodeling in endothelial cells. The use of new microscopy techniques including the use of novel FRET-based tension sensors [68, 252, 253] as well as kinase and GTPase biosensors [254–257] will undoubtedly allow us to assess the extent of localized and temporally limited changes in junctional association in the context of a forming gap at the specific locations where intercellular adhesion is being affected. Combining these assays with the newly developed phosphospecific antibodies with much improved epitope specificity [81, 89–91] will enable us to correlate in time and space the level of VE-cadherin phosphorylation with its association with catenins in the endothelial cell response to edemagenic stimuli and to leukocyte diapedesis.
